# Circulating ghrelin level is higher in HNF1A–MODY and GCK–MODY than in polygenic forms of diabetes mellitus

**DOI:** 10.1007/s12020-015-0627-5

**Published:** 2015-05-19

**Authors:** Natalia Nowak, Jerzy Hohendorff, Iwona Solecka, Magdalena Szopa, Jan Skupien, Beata Kiec-Wilk, Wojciech Mlynarski, Maciej T. Malecki

**Affiliations:** Department of Metabolic Diseases, Jagiellonian University Medical College, 15 Kopernika Street, 31-501 Krakow, Poland; Section on Genetics and Epidemiology, Joslin Diabetes Center, Boston, MA USA; University Hospital, Krakow, Poland; Department of Pediatrics, Oncology, Hematology and Diabetology, Medical University of Lodz, Lodz, Poland

**Keywords:** GCK–MODY, HNF1A–MODY, T1DM, T2DM, Ghrelin

## Abstract

Ghrelin is a hormone that regulates appetite. It is likely to be involved in the pathophysiology of varying forms of diabetes. In animal studies, the ghrelin expression was regulated by the hepatocyte nuclear factor 1 alpha (HNF1A). Mutations of the *HNF1A* gene cause maturity onset diabetes of the young (MODY). We aimed to assess the circulating ghrelin levels in HNF1A–MODY and in other types of diabetes and to evaluate its association with *HNF1A* mutation status. Our cohort included 46 diabetic *HNF1A* gene mutation carriers, 55 type 2 diabetes (T2DM) subjects, 42 type 1 diabetes (T1DM) patients, and 31 glucokinase (*GCK*) gene mutation carriers with diabetes as well as 51 healthy controls. Plasma ghrelin concentration was measured using the immunoenzymatic assay with polyclonal antibody against the C-terminal fragment of its acylated and desacylated forms. Ghrelin concentrations were 0.75 ± 0.32, 0.70 ± 0.21, 0.50 ± 0.20, and 0.40 ± 0.16 ng/ml in patients with HNF1A–MODY, GCK–MODY, T1DM, and T2DM, respectively. The ghrelin levels were higher in HNF1A–MODY and GCK–MODY than in T1DM and T2DM (*p* < 0.001 for all comparisons) but lower than in non-diabetic controls (1.02 ± 0.29 ng/ml, *p* < 0.001 for both comparisons). In the multivariate linear model, the differences between both MODY groups and common diabetes types remained significant. Analysis by a *HNF1A* mutation type indicated that ghrelin concentration is similar in patients with different types of sequence differences. Plasma ghrelin level is higher in HNF1A–MODY and GCK–MODY than in the common polygenic forms of diabetes.

## Introduction

Monogenic diabetes accounts for only a small portion of diabetes cases; however, a proper differential diagnosis usually brings substantial clinical benefits for the subjects affected by a single gene form of this disease. Monogenic diabetes is caused by a mutation in one of about a dozen of genes. One of the most common is the defect of the *HNF1A* (Hepatic Nuclear Factor 1A) gene, that accounts for approximately a half of all cases of maturity onset diabetes of the young (MODY). MODY is characterized by early disease onset and an autosomal dominant mode of inheritance [1]. HNF1A is a transcription factor that controls β-cell development, mass, and function. It is also expressed in digestive tract, liver, and kidneys participating in the regulation of a wide number of genes [2, 3].

In the recent studies, HNF1A protein was shown as an upstream regulator of several neuroendocrine peptides. Among them, ghrelin, a peptide hormone the amino acid sequence of which is highly conserved among mammals, was identified as one of the targets [4]. Ghrelin is secreted mostly by stomach but is also widely expressed in the other locations, including pancreatic cells. It stimulates appetite and regulates secretion of other hormones, such as growth hormone, glucagon, and insulin [5]. Ghrelin is a non-glycosylated peptide, which is present in the bloodstream in two major molecular forms: desacylated and acylated. The desacylated molecular form accounts for 90 % of the circulating ghrelin. Acylated ghrelin was considered to be the only metabolically active form of ghrelin peptide acting as a mediator in metabolic, hormonal, and inflammation processes in humans [6]. However, recent studies have showed that desacylated ghrelin is also functionally active; however, its metabolic role has not yet been defined [7–10].

In vitro studies have shown that HNF1A interacts with specific binding sites of the ghrelin gene promoter and suppresses hormone expression [11]. In the experimental animals, ghrelin mRNA level was increased by approximately five-fold in homozygous *HNF1A* knock-out mice as compared to the wild type [11, 12]. This was further followed by five-fold higher concentration of the total and active forms in serum and, interestingly, by a subsequent decrease of insulin level. Consistent with these findings, a targeted silencing of *HNF1A* gene expression in the pancreatic endocrine cell line induced ghrelin gene transcript [12].

Taken together, the body of evidence shows that HNF1A acts as a repressor of ghrelin secretion through a direct effect on its promoter. So far, the ghrelin level in human *HNF1A* gene mutation carriers or other MODY subjects has not been examined, while data from type 1 (T1DM) and type 2 diabetes (T2DM) are limited. The primary aim of this study was to compare plasma ghrelin level in HNFA1–MODY with glucokinase (GCK)–MODY, T1DM, and T2DM subjects as well as non-diabetic healthy individuals. Additionally, we evaluated plasma ghrelin as a biomarker of *HNF1A* mutation status.

## Materials and methods

### Study population

The studied group included 46 HNF1A–MODY individuals, 31 *GCK* gene mutation carriers with diabetes, 55 T2DM subjects, and 42 T1DM patients. In addition, 51 healthy non-carrier individuals were recruited from the families of patients with MODY, as the reference group. All MODY cases had a heterozygous loss-of-function mutation either in the *HNF1A* or *GCK* gene identified by direct DNA sequencing. For further analysis, the mutations within HNF1A gene were classified with respect to their type as either protein-changing (related to a missense change of amino acid) or truncating (resulting in a premature stop codon) mutations and according to the affected functional domain (dimerization/DNA-binding domain, or transactivation domain) [13, 14]. We included patients with clinical diagnosis of T2DM only if they had a disease detected below the age of 45 years, so that rough age matching could be performed and had no insulin treatment for at least 2 years after the initiation of pharmacotherapy, which were the criteria we used in our previous research to differentiate T2DM patients from subjects with autoimmune diabetes [15]. Subjects with T1DM were ascertained if at diagnosis they had typical clinical symptoms, insulin therapy requirement from the beginning of the disease, and diabetes diagnosed below 30 years of age. For all study subjects, we collected data on their clinical characteristics and determined ghrelin levels in plasma specimens. We excluded subjects with chronic kidney disease (defined as CKD-EPI GFR <60 ml/min/1.73 m^2^), individuals on steroid therapy, and pregnant women. The protocol of the study was approved by the Bioethical Committee of the Jagiellonian University and all subjects gave written informed consent.

### Ghrelin measurement

We used ghrelin specific immunoenzymatic assay (Phoenix Pharmaceuticals, Belmont, CA) that recognizes the C-terminal part of ghrelin peptide chain. As the literature suggested that sample pretreatment might influence the N-terminal fragment stability, the C-terminal based kit was used [16]. This one-site assay determines both intact ghrelin and some of its more stable breakdown products. The lower and upper limits of quantification for this assay were 0.11 and 1.60 ng/ml, respectively. The other validation parameters of the assay such as lower limit of quantification, linearity of the dilution, and cross-reactivity have already been reported by the manufacturer. The measurements were performed in specimens collected after at least 8-h fasting. Venous blood was drawn into EDTA tubes. Samples were stored at 4 °C during the collection period and then centrifuged at 4 °C. The plasma was separated into aliquots and then stored at −40 °C until assayed. All assays included control samples which were collected at the beginning of the study, transferred to aliquots, and used to test for assay variability. Based on these, the intra-assay and inter-assay coefficients of variation (CV) were 6 and 22 %, respectively. The diabetic subgroups were represented equally in each plate.

### Statistical analysis

The computations were made with SAS, Version 9.3 (SAS Institute, Cary, NC), and MedCalc, Version 12.1.4 (MedCalc Software, Mariakerke, Belgium). P values for testing differences across groups were calculated with one-way ANOVA followed by Tukey’s post hoc test. The comparison of categorical variables was done by *χ*^2^ test. We assessed if MODY predicted ghrelin concentration using linear regression models, with type of diabetes as the explanatory variable. The clinical factors (age of examination, diabetes duration, BMI, gender, treatment with insulin, HbA1c concentration) were individually tested as predictors and then were analyzed in multiple linear regression models with backward selection of covariates. The variables kept in the model were further used as basic model for ghrelin at testing differences between various diabetic subgroups; group presented as ordinal variable was added into general linear model, to test independence from other ghrelin predictors. The potential of co-linearity was assessed by variance inflation factors (VIF), with VIF less than 10 considered as acceptable. To control for the extent of degradation of the analyte, we also included length of storage, defined as the time between blood collection and assay determination, as a covariate in additional regression analysis. Including this variable did not affect the study results. Diagnostic performance (i.e., the ability of ghrelin to identify HNF1A–MODY and GCK–MODY) was assessed using the receiver operating characteristics (ROC) curve. The standard error (SE) of the area under the ROC curve (AUC) and 95 % confidence intervals (95 % CI) were calculated using the method described by DeLong et al. [17]. All tests were two-tailed, and *p* value <0.05 was considered significant.

## Results

The study groups’ clinical characteristics and biochemical measurements are summarized in Table 1. No significant differences were observed between the HNF1A–MODY, GCK–MODY, and T1DM in terms of age, sex distribution, and body mass index (BMI). Patients in T2DM group were significantly older, more obese, and had later diabetes onset than MODY subjects, which is in line with the way the groups were defined. Also, patients with common form of diabetes demonstrated worse glycemic control than individuals with MODY, as assessed by the fasting glucose and HbA1c levels.

**Table 1:** Clinical characteristics of patients and controls without diabetes

Characteristics	HNF1A–MODY	T1DM	T2DM	GCK–MODY	ND	*P* value
No. of individuals	46	42	55	31	51	NA
Female/male	27/19	21/24	19/36	17/13	28/23	0.09^&^
Age at examination (years)	31.6 (27.1–36.1)	31.5 (29.2–33.9)^^#^	53.5 (50.5–55.7)^^#^	35.9 (28.7–43.2)	38.7^	<0.001*
Diabetes duration (years)	11.2 (7.7–14.7)	8.9 (6.8–10.9)^^#^	13.1 (10.6–15.4)^^#^	5.5 (2.6–8.4)	NA	<0.001*
BMI (kg/m^2^)	22.9 (21.8–24.2)	23.6 (22.6–24.7)	31.1 (30.1–33.3)^^#^	24.4 (22.6–26.2)	23.3 (22.4–26.2)	<0.001*
HbA1c (%)	6.7 (6.3–7.1)	8.1 (7.5–8.6)^^#^	8.7 (8.3–9.2)^#	6.7 (6.2–7.1	NA	<0.001*
Fasting glucose (mmol/L)	6.4 (5.6–7.1)	7.7 (7.2–8.5)	8.74 (8.3–9.3)^^#^	6.1 (5.1–6.9)	NA	<0.001*
% on insulin	36 %	100 %	63 %	26 %	NA	<0.001^&^
% on OHA	62 %	0 %	27 %	23 %	NA	<0.001^&^
Ghrelin (ng/ml)	0.75; 0.73; (0.66–0.86)	0.50; 0.47 (0.44–0.57)	0.40; 0.40 (0.36–0.45)	0.7; 0.66 (0.62–0.78)	1.02; 1.01 (1.01–1.14)	<0.001*

Data are expressed as means and 95 % confidence intervals for the mean, except treatment, and sex proportions. Ghrelin concentration was reported as mean, median, and 95 % for the mean

*N* number of individuals, *BMI* body mass index, *HbA1c* Hemoglobin A1c, *OHA* oral anti-hyperglycemic agents

* One-way ANOVA

^&^ *χ* ^2^ test

^ *P* value based on Tukey post hoc test for the difference versus HNF1A–MODY <0.05

^#^ *P* value based on post hoc Tukey test for testing the difference versus GCK–MODY <0.05

**Fig. 1:**
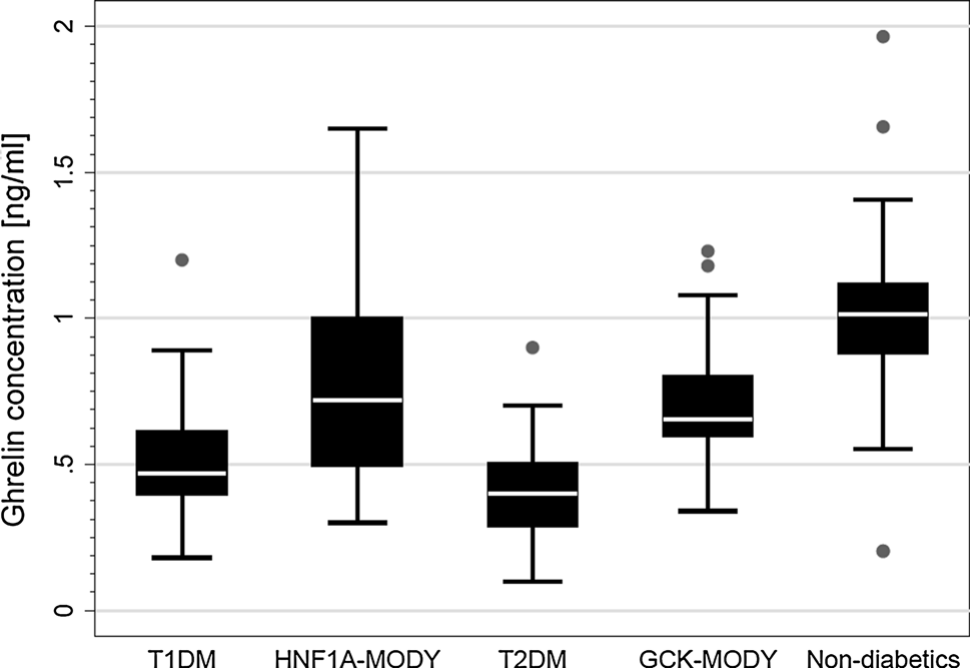
The distribution of crude values of ghrelin level in the study groups

The distribution of crude values of ghrelin in the study groups is shown in Fig. 1. Mean concentration in HNF1A–MODY subjects was 0.75 ng/ml (SD = 0.32 ng/ml). It was approximately twofold higher than in the T2DM group (0.40 ± 0.16 ng/ml; *p* < 0.001) and 50 % higher than in the T1DM group (0.50 ± 0.20 ng/ml; *p* < 0.001). Ghrelin level was lower in all diabetic subjects than in the healthy controls (1.02 ± 0.29 ng/ml, *p* < 0.001). Ghrelin level was also lower in HNF1A–MODY and in GCK–MODY as compared to non-diabetics (*p* < 0.001 for both comparisons). No measurable differences were observed between the crude ghrelin concentrations in HNF1A–MODY and GCK–MODY (0.70 ± 0.21 ng/ml; *p* = 0.36), that similarly presented higher levels in comparison to T1DM (*p* < 0.001) and T2DM (*p* < 0.001).

In the HNF1A–MODY group, ghrelin level significantly correlated with age at examination (*ρ* = −0.39, *p* = 0.008), diabetes duration (*ρ* = −0.30, *p* = 0.05), and was marginally correlated with BMI (*ρ* = −0.25, *p* = 0.1), whereas in GCK–MODY, there was a significant correlation with age (*ρ* = −0.41, *p* = 0.02). In the type T2DM group, there was significant correlation only with BMI (*ρ* = −0.44, *p* < 0.001). No significant correlation was observed, in the diabetic subgroups, for HbA1c, and fasting glucose level. In the analyses conducted in pooled diabetic groups, ghrelin level correlated inversely with BMI (*ρ* = −0.37; *p* < 0.001), age at the examination (*ρ* = −0.31; *p* < 0.001), and with the HbA1c level (*ρ* = −0.31; *p* < 0.001). Sex influenced ghrelin level in all diabetic groups combined, as well as in a separate analysis of HNF1A–MODY individuals (0.51 ± 0.26 vs. 0.64 ± 0.29 ng/ml; *p* = 0.001 for males and females in combined diabetic cohort and 0.62 ± 0.25 vs. 0.82 ± 0.37 ng/ml; *p* = 0.04 in the HNF1A–MODY, respectively). In multivariate linear regression (*R*^2^ = 25 %), only significant predictors remained BMI (*p* = 0.002) and age at examination (*p* = 0.001). When significance (*α* value) was set up at 0.1 (instead of 0.05) during testing of predictors and analyzing in multiple linear regression model, insulin treatment status was additionally kept in the model. However, the only significant variables were BMI and age at examination.

In a general linear model (*R*^2^ = 36 %) applied in polled diabetic groups, the differences between HNF1A–MODY and T1DM and between GCK–MODY and T1DM remained significant; their pattern and magnitude remained unchanged to that in univariate analysis (*p* < 0.001 for both comparisons). Group was the strongest independent variable associated with ghrelin, the next one being age (*p* = 0.004), and BMI (*p* = 0.01).

**Fig. 2:**
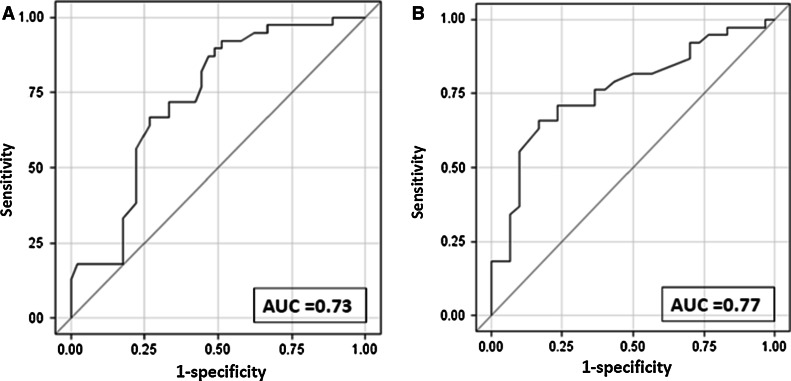
ROC curves illustrating discriminative performance of plasma ghrelin to distinguish between diabetic subgroups. **a** HNF1A MODY and type 1 diabetes,** b** GCK MODY and type 1 diabetes

The ROC curves illustrating ghrelin capacity in distinguishing both examined MODY types and T1DM are presented in Fig. 2. The discriminative accuracy, as expressed by AUC of ghrelin between HNF1A–MODY and T1DM, was 0.73 (95 % CI 0.63; 0.84) with the corresponding sensitivity and specificity of 74 % (95 % CI 57.2; 85) and 66.7 % (95 % CI 49.3–98.90), respectively. For GCK–MODY and T1DM, a slightly better result was obtained (AUC = 0.77; 95 % CI 0.66; 0.89).

With the adjustment for BMI and age at examination the ghrelin concentrations in T2DM and both MODY groups had similar pattern but the differences were reduced by approximately 50 % as compared with crude estimates (*p* = 0.007, and *p* = 0.005 for HNF1A–MODY and GCK–MODY, accordingly). The covariate-adjusted AUC showed a weak distinction between HNF1A–MODY and T2DM (AUC = 0.70; 95 % CI 0.51–0.88), and only a slightly better for GCK–MODY and T2DM (AUC = 0.77; 95 % CI 0.58–0.97).

We also examined the influence of the type and localization of *HNF1A* mutation on ghrelin concentration. Among *HNF1A* mutations, 22 were classified as truncating and 24 as missense changes. While none were assigned to DNA-binding domains, 26 were assigned to dimerization, and 20 were assigned to transactivation domains of the peptide. Analysis by mutation type indicated that ghrelin concentration was circa 15 % (0.11 ng/ml) higher in HNF1A–MODY patients with protein-changing mutations than those with truncating mutations; however, the difference did not reached significance (*p* = 0.3). No measurable difference in ghrelin concentration was found between mutations located in DNA-binding and transactivation domains (*p* = 0.97).

## Discussion

Here, for the first time, we examined the plasma ghrelin levels across several types of diabetes and in non-diabetic controls. Our results showed that circulating ghrelin concentration may, at least to some degree, depend on the etiology of diabetes.

Ghrelin plays an important regulatory role in metabolism, particularly in appetite control [18]. Additionally, ghrelin influences insulin secretion, glucose homeostasis, and adipogenesis processes [5, 19]. In animal models, ghrelin expression and its peptide concentration were higher in mice with homozygous knockout of the *HNF1A* gene as compared to the wild type. However, we were not able to confirm this in the study on human subjects, as, contrary to the initial hypothesis of our project, ghrelin level was lower in HNF1A–MODY caused by a heterozygous loss-of-function mutation than in the non-diabetic individuals. On the other hand, it was higher in HNF1A–MODY patients (as well as GCK–MODY individuals) than in both common forms of diabetes. Interestingly, in our study, ghrelin level did not differ between HNF1A–MODY and GCK–MODY patients. For T2DM, this difference was partly dependent on the clinical characteristics of a group chosen for the comparison. In our study and previously published reports, ghrelin correlated negatively with BMI and age in healthy subjects as well as diabetic individuals [20, 21]. Also, low ghrelin level in T2DM as compared to non-diabetic controls has been earlier attributed to the presence of chronic inflammation, insulin resistance, and hyperinsulinemia [6, 22]. The observed negative correlation between ghrelin and age is similar to that reported from other studies in diabetic and healthy individuals, and so is consistent ghrelin age-related decline. Nevertheless, although T2DM patients were significantly older than subjects from the other groups, the observed differences persisted, although diminished, even after adjustment for age at study entry. So far, very limited data have been published on ghrelin concentrations in T1DM patients. However, one study had shown that ghrelin level was lower in the newly diagnosed T1DM children than in the non-diabetic individuals [23]. Likewise the T1DM children lacked proper ghrelin response to a meal [24]. We consider that differences in levels of glycemic control are among the factors potentially contributing to the observed variability in ghrelin concentration between the examined groups. Some differences might be also related to monogenic alterations in GCK and HNF1A proteins, acting through different, not yet identified, mechanisms.

An interesting facet of ghrelin pathophysiology is related to its potential contribution to the elevated glucose level in patients with diabetes and to its putative therapeutic use. For example, it was shown that ghrelin contributed to the *HNF1A* loss-related hyperglyceamia in rodents and the ghrelin receptor blockade in *HNF1A* knockout mice led to a recovery of diabetic symptoms [6]. It has also been documented that ghrelin reduces glucose-induced insulin secretion in healthy human subjects [19, 25]. The mechanism of this phenomenon may involve a suppression of insulin secretion in which ghrelin either acts directly via activation of the β-cell growth hormone secretagogue receptor (GHS-R1a) or indirectly through interaction with the vagus nerve [26]. In our study, ghrelin was unrelated to any parameters of glycemic control in stratified analysis, while some other expected associations were present. The potential modification of glycemic control by ghrelin in patients with diabetes as well as the putative glucose-lowering mechanism related to inhibition of its pathway requires further investigation using a different study design than ours. As acylated and desacylated ghrelin isoforms influence glucose homeostasis in opposite directions, their proportion should be assessed in future studies [7–9].

Currently, most of the MODY patients remained misdiagnosed as T1DM or T2DM. A proper differential diagnosis brings a clinical benefit to the affected individuals and their families. For example, it helps tailor more personalized treatment and define prognosis in the family. Thus, it would be beneficial to identify cheap and easy-to-use non-genetic markers that could be applied for screening a wide number of subjects for different MODY subtypes [27]. There were a few such promising biomarkers examined over the last years, such as hs-CRP, 1,5-anhydroglucitol, apolipoprotein M, and some others; nevertheless, none of them have entered widely into clinical use [28, 29]. The crude ghrelin capacity for distinguishing MODY from T1DM and T2DM was moderate; additionally, in T2DM it was partially dependent on the variability in basic clinical covariates that are considered in diagnostic algorithm. Based on the results of our study, we are not able to recommend ghrelin as a robust parameter in differential diagnosis of MODY.

Finally, we did not identify any convincing evidence for the possible association of type of *HNF1A* mutation with the ghrelin level, although the concentration of examined hormone tended to be higher in patients with missense protein-changing *HNF1A* mutations as compared to the ones with truncating sequence differences. Interestingly, the type of *HNF1A* mutation, as reported earlier, seems to modify levels of other circulating biomarkers, such as hs-CRP and DG9-glycan index levels, with missense mutations being related to the lower level of examined particles [29, 30]. These results could probably be explained by a dominant-negative effect, which was reported for some *HNF1A* mutations. We also did not find any evidence for the earlier reported relationship between the localization of gene sequence differences on ghrelin concentration [29, 30]. This may be explained by a modest effect of *HNF1A* on ghrelin in human subjects, small number of study subjects in subgroups used for comparison (a shortcoming of the current study), or by presence of the unidentified confounding factors [31, 32].

In summary, plasma ghrelin level seems to depend on the etiology of diabetes and is higher in HNF1A–MODY and GCK–MODY than in both common polygenic forms of diabetes. However, it does not seem to be a good biomarker in differential diagnosis of diabetes subtypes.

## Abbreviations

BMI: Body mass index
GCK: Glucokinase
GHS-R1a: Growth hormone secretagogue receptor 1a
HbA1c: Hemoglobin A1c
HNF1A: Hepatic nuclear factor 1A
MODY: Maturity onset diabetes of the young
